# Excelencia científica en la periferia y nichos de experiencia, particularidades de la constitución de la microbiología en Centroamérica

**DOI:** 10.1590/S0104-59702023000100021

**Published:** 2023-05-15

**Authors:** Ligia María Peña Torres

**Affiliations:** i Universidad Centroamérica. Managua – Nicaragua petoli.5701@gmail.com

La obra (Viales Hurtado, Rodríguez Sánchez, 2021) es el resultado del trabajo de un grupo interdisciplinario de investigadores, agrupados en la Red de Historia de la Microbiología en Contexto Global, Siglos XIX-XXI. Los académicos se enfocaron en el análisis comparado de dicha temática en el contexto global, con especial referencia al caso de Iberoamérica. Resulta muy interesante la visión de esta Red al plantearse realizar investigaciones con perspectiva histórica e integrando la visión que ofrecen los análisis de la historia social, la historia tecnocientífica, los estudios sociales de la ciencia, la historia de la salud y la enfermedad, entre otros enfoques teóricos, para analizar los procesos de institucionalización, profesionalización y desarrollo de la microbiología en Iberoamérica.

Para el grupo de académicos de la Red, hacer historia de la microbiología en contexto global significó colocar América Latina y España en un plano de acción e interacción mucho más amplio, donde la relación entre microorganismos, disciplinas científicas, salud, enfermedad, epidemias y sociedad se constituyeron en ejes transversales de investigación, enfocándose en un tipo de historia democratizante, que deja claro el papel de los centros principales de investigación en el contexto global, pero también el intercambio con el desarrollo científico que se hace desde Iberoamérica.

La obra se estructura con un amplio espectro de temáticas que piensan y escriben la historia de la microbiología en Iberoamérica, en comunicación con la historia global. Los estudios de casos dan cuenta de las particularidades que revistió en España, Argentina y Costa Rica el surgimiento de la microbiología como ciencia, campo de formación y de investigación.

La relación entre microbiología y epidemiología durante el siglo XX en España es objeto de análisis del capítulo primero donde se deja claro que la trayectoria de esta relación se construyó a partir de la antigua conexión entre salud pública y bacteriología. Refiriéndose a Argentina, realiza una exploración detallada del proceso de creación del Instituto Bacteriológico de Argentina, enfatiza en la dinámica de producción de conocimientos y técnicas, y los diferentes sujetos sociales que intervienen en la introducción del paradigma de la bacteriología entre finales del siglo XIX e inicios del XX.

Entre 1975 y 2019, en el campo de la constitución de la microbiología como ciencia en Costa Rica y por medio del estudio de las principales instituciones de investigación, y las personas participantes en redes de investigación, y proyectos de investigación registrados en la base de datos de investigación de la Universidad de Costa Rica (UCR), la construcción de redes sociotécnicas para la producción de conocimiento científico ocupa una característica central de las reflexiones sobre la constitución de la microbiología como ciencia en Costa Rica. Asi mismo, resulta interesante el recorrido por los diversos aspectos del desarrollo histórico de la cooperación internacional en el Instituto Clodomiro Picado, cuyas metas y acciones muestran una clara vocación hacia la internacionalización desde los primeros años de su existencia.

Para Centroamérica, desde el punto de vista de la historia de las ciencias y la salud, los capítulos más sugerentes son el dos y el tres. En el capítulo dos se conoce “El surgimiento de la microbiología médica como campo de conocimiento, el contexto global y los determinantes para su surgimiento en Costa Rica, 1870-1930”, y el capítulo tres trata “La constitución de la microbiología como disciplina y campo de formación e investigación en Costa Rica, 1870-1957”. Ambos condensan los elementos históricos y características generales y particulares de la dinámica del surgimiento de la microbiología en Costa Rica.

Dos elementos llaman la atención: la aproximación al objeto de estudio y su aporte a la historia de las ciencias y la salud en Centroamérica y Costa Rica.

## Los intercontextos en el análisis histórico desde la historia global

El acercamiento al objeto de estudio se realiza atraves de la categoría “intercontextos”, que forma la columna vertebral del análisis y narrativa histórica. Ronny Viales Hurtado (2018) se pregunta ¿cómo realizar una (re)lectura de la historia de Centroamérica en el gran contexto de la historia mundial y desde la historia global? Su propuesta metodológica de análisis es utilizar la noción de intercontextos. Ésta se refiere a los diferentes contextos relacionados que inciden sobre, o sobre los que inciden, las problemáticas de investigación histórica, tal como la que se estudia en esta obra. Éstos pueden ser factores explicativos, mecanismos causales, ideas, discursos, conceptos y subjetividades que circulan y/o sirven como referentes para ampliar las posibilidades de descripción, interpretación o explicación de hechos históricos. Potencian la posibilidad de pensar los hechos históricos glo-cales, y están en sintonía con la historia mundial y con la perspectiva de la historia global.

Esta perspectiva metodológica facilita perfilar en los textos una diversidad de elementos, variables, ideas, sujetos sociales nacionales e internacionales, instituciones locales, y externas, politicas públicas, el papel del estado; que constituirían los intercontextos, que se articulan y/ o conjugan, y que al mismo tiempo marcan los límites que dichos componentes tuvieron en el proceso de institucionalización de la microbiología en Costa Rica como disciplina, campo de formación y de investigación entre 1870 y mediados del siglo XX.

Resulta interesante en el análisis cómo los autores subrayan en cada contexto cómo estos contribuyen y/o marcan los límites a la compresión del objeto estudio. No menos atrayente, resulta conocer que Costa Rica aprovechó de manera creativa todas las coyunturas y oportunidades a nivel global para promover en estos espacios la microbiología como ciencia, campo de formación e investigación.

## Excelencia científica en la periferia y nichos de experiencia en la de configuración de la microbiología en Costa Rica

Marcos Cueto (1989) postuló la noción de “excelencia científica desde la periferia”. El centro de esta noción se refiere a que no toda la ciencia que se hace en los paises atrasados es marginal al acervo mundial del conocimiento, y que el trabajo científico desarrollado en estos paises tiene sus propias reglas que deben ser entendidas no como síntomas de atraso o modernidad, sino como parte de su propia cultura y de las interacciones con la ciencia internacional.

Siguiendo este postulado, los autores analizan y documentan, a traves de diversas fuentes bibliográficas y archivísticas, todos y cada uno de los elementos, procesos, variables, instituciones nacionales e internacionales, actores sociales, saberes y corrientes de pensamiento que circulaban tanto a nivel global como en América Latina y Centroamérica en el periodo en estudio. Destacan los múltiples contextos que interactuaban y/o se articulaban de manera creativa, con las agendas locales ya establecidas en Costa Rica, en el campo de la salud pública, las politicas de salud, el desarrollo de la medicina tropical y en la naciente constitución de la microbiología como disciplina, y como campo de formación e investigación. Estas evidencias demuestran el hecho que a pesar que los paises perifericos, como los de la región centroamericana, situados de manera desventajosa en la división internacional del trabajo en el campo de la salud, ya tenían, como Costa Rica, una importante presencia en el campo científico, y, por consiguiente, participaba de la excelencia científica desde la periferia.

## Nichos de experiencias

Marcos Cueto y Steven Palmer (2015) consideran que la bacteriología en América Latina se insertó tempranamente con las agendas estatales nacionales lo que permitió la generación de “nichos de experiencias”, sobre todo en el campo de las enfermedades tropicales. En este sentido se destacan para el caso de Costa Rica, cómo las investigaciones del doctor Carlos Duran, en 1847, acerca de la anquilostomiasis, contribuyeron a mejorar desde el punto de vista curativo el tratamiento para esta enfermedad.

En suma, la obra incluye un valioso conjunto de estudios sobre el surgimiento de la microbiología en Iberoamérica desde diferentes perspectivas analíticas. Particularmente, la experiencia costarricense aporta elementos metodológicos, fuentes documentales novedosas y una perspectiva de análisis fundamentada en la perspectiva de la historia global que permite destacar los diferentes contextos y sus relaciones con el objeto de estudio, situando la obra como un referente sumamente importante para futuras investigaciones sobre la historia de las ciencias y de la salud en Centroamérica.
